# Supplementary material for: Identification of Freezing-Responsive microRNAs and Their Targets in Chinese Jujube by Small RNA and Degradome Sequencing
Source: Int J Mol Sci. 2026 Sep 4;27(17):7912. doi: 10.3390/ijms27177912 (PMC13566890; doi:10.3390/ijms27177912)
Supplement: Supplementary file 1 [file ijms-27-07912-s001.zip › Supplementary Material S2/novel_miR_203.pdf]

| | |
|---|---|
| Provisional ID | : novel_miR_203 |
| Score total | : 0.6 |
| Score for star read(s) | : -1.3 |
| Score for read counts | : 0 |
| Score for mfe | : 0.3 |
| Score for randfold | : 1.6 |
| Score for cons. seed | : |
| Total read count | : 332 |
| Mature read count | : 321 |
| Loop read count | : 0 |
| Star read count | : 11 |

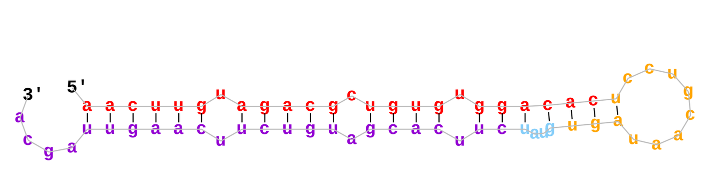


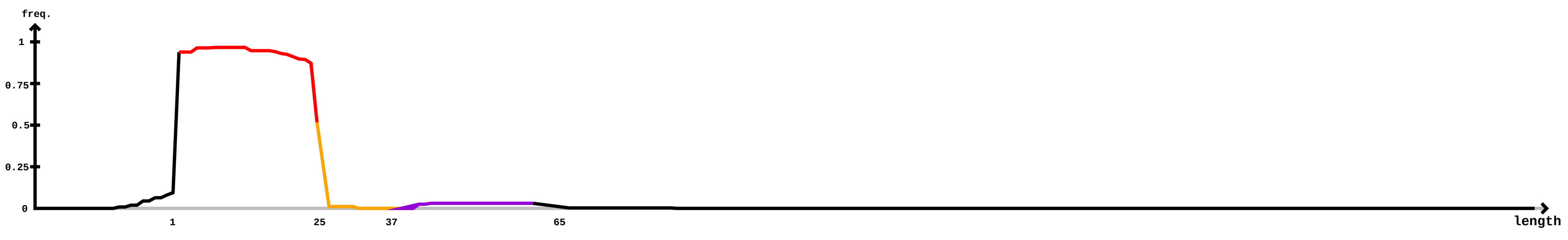


Mature Star

```
5'- guuuggcuuaggugagaaugcuaacuuguagacgcuguguggacacuccugcaauaguguaucuucacgaugucuucaaguuagcauccucuuuuuugcaucauguauauauacauauauauauauauauauauauauauaugugcauccgacuggggucucugggcgnnnnnnnnnnnnnnnnnnnnnnnnnnnnnnnnnnnnnnnnnnnnnnnnnnnnnnnnnnnnnnnnnnnnnnnnnnnnnnnnnnnnnnnnnnnnnnnnnnnnnnnnn -3'  obs
    guuuggcuuaggugagaaugcuaacuuguagacgcuguguggacacuccugcaauaguguaucuucacgaugucuucaaguuagcauccucuuuuuugcaucauguauauauacauauauauauauauauauauauauauaugugcauccgacuggggucucugggcgnnnnnnnnnnnnnnnnnnnnnnnnnnnnnnnnnnnnnnnnnnnnnnnnnnnnnnnnnnnnnnnnnnnnnnnnnnnnnnnnnnnnnnnnnnnnnnnnnnnnnnnnn       exp
    .....(((((((.(((.(((((((((((.(((((.((((.(((((((.......))))..))).)))).))))).))))))))))).)))......(((.((((((((((((.(((((....))))).)))))))))))))))((((.....)))).)))))))................................................................................................................  reads  mm  sample
    ............ugagaaugcuaacuuguagacg..................................................................................................................................................................................................................................................  1  0  JCK-1
    ............ugagaaugcuaacuuguagacg..................................................................................................................................................................................................................................................  2  0  JCK-3
    ..............agaaugcuaacuuguagacg..................................................................................................................................................................................................................................................  2  0  LT-2
    ..............agaaugcuaacuuguagacg..................................................................................................................................................................................................................................................  1  0  LT-3
    ..............agaaugcuaacuuguagacg..................................................................................................................................................................................................................................................  1  0  LT-1
    ................Caugcuaacuuguagacgcugug.............................................................................................................................................................................................................................................  1  1  JCK-1
    ................Caugcuaacuuguagacgcugug.............................................................................................................................................................................................................................................  2  1  JCK-3
    ................aaugcuaacuuguagacgcugugu............................................................................................................................................................................................................................................  2  0  LCK-3
    ................aaugcuaacuuguagacgcuguguA...........................................................................................................................................................................................................................................  4  1  LT-2
    ..................ugcuaacuuguagacgcugug.............................................................................................................................................................................................................................................  1  0  LT-1
    ..................ugcuaacuuguagacgcugugugg..........................................................................................................................................................................................................................................  5  0  LCK-3
    ..................ugcuaacuuguagacgcuguguggU.........................................................................................................................................................................................................................................  1  1  LT-3
    ....................cuaacuuguagacgcugu..............................................................................................................................................................................................................................................  2  0  JCK-2
    ....................cuaacuuguagacgcuguguggacac......................................................................................................................................................................................................................................  1  0  JCK-1
    ....................cuaacuuguagacgcuguguggacac......................................................................................................................................................................................................................................  2  0  LT-2
    ....................cuaacuuguagacgcuguguggacac......................................................................................................................................................................................................................................  1  0  JCK-2
    .....................uaacuuguagacgcuguguggaca.......................................................................................................................................................................................................................................  1  0  LT-2
    .....................uaacuuguagacgcuguguggacac......................................................................................................................................................................................................................................  4  0  LT-2
    ......................aacuuguagacgcugugug...........................................................................................................................................................................................................................................  1  0  LT-1
    ......................aacuuguagacgcuguguggac........................................................................................................................................................................................................................................  1  0  JCK-3
    ......................aacuuguagacgcuguguggac........................................................................................................................................................................................................................................  2  0  JCK-2
    ......................aacuuguagacgcuguguggaca.......................................................................................................................................................................................................................................  5  0  LT-2
    ......................aacuuguagacgUuguguggaca.......................................................................................................................................................................................................................................  1  1  JCK-1
    ......................aacuuguagacgcuguguggaca.......................................................................................................................................................................................................................................  5  0  LT-1
    ......................aacuuguagacgUuguguggaca.......................................................................................................................................................................................................................................  1  1  JCK-3
    ......................aacuuguagacgcuguguggaca.......................................................................................................................................................................................................................................  19  0  JT-1
    ......................aacuuguagacgcuguguggaca.......................................................................................................................................................................................................................................  18  0  JCK-1
    ......................aacuuguagacgcuguguggaca.......................................................................................................................................................................................................................................  21  0  JCK-3
    ......................aacuuguagacgcuguguggaca.......................................................................................................................................................................................................................................  32  0  JCK-2
    ......................aacuuguagacgcuguguggaca.......................................................................................................................................................................................................................................  6  0  LT-3
    ......................aacuuguagacgcuguguggaca.......................................................................................................................................................................................................................................  19  0  LCK-3
    ......................aacuuguagacgcuguguggacac......................................................................................................................................................................................................................................  35  0  JCK-2
    ......................aacuuguagacgcuguguggacac......................................................................................................................................................................................................................................  24  0  JCK-1
```

```
                      Mature                                  Star
guuuggcuuaggugagaaugcuaacuuguagacgcuguguggacacuccugcaauaguguaucuucacgaugucuucaaguuagcauccucuuuuuugcaucauguauauauacauauauauauauauauauauaugugcauccgacuggggucucugggcgnnnnnnnnnnnnnnnnnnnnnnnnnnnnnnnnnnnnnnnnnnnnnnnnnnnnnnnnnnnnnnnnnnnnnnnnn
......................aacuuguagacgcuguguggacac..........................................................   37   0   LCK-3
......................aacuuguagacgcuguguggacac..........................................................   2    0   LT-3
......................aacuuguagacgcuguguggacac..........................................................   27   0   JCK-3
......................aacuuguagacgcuguguggacac..........................................................   7    0   LT-2
......................aacuuguCgacgcuguguggacac..........................................................   1    1   LCK-3
......................aacuuguagacgcuguguggacac..........................................................   4    0   LT-1
......................aaNuuguagacgcuguguggacac..........................................................   1    1   LT-2
......................aacuuguagacgcuguguggacacu.........................................................   4    0   JT-2
......................aacuuguagacgcuguguggacacC.........................................................   1    1   JT-3
......................aacuuguagacgcuguguggacacu.........................................................   3    0   JT-3
......................aacuuguagacgcuguguggacacA.........................................................   1    1   JCK-1
......................aacuuguagacgcuguguggacacu.........................................................   6    0   JCK-3
......................Cacuuguagacgcuguguggacacu.........................................................   1    1   JCK-3
......................aacuuguagacgcuguguggacacu.........................................................   9    0   JCK-2
......................aacuuguagacgcuguguggacacA.........................................................   1    1   JCK-2
......................aacuuguagacgcuguguggacacu.........................................................   3    0   LT-3
......................aacuuguagacgcuguguggacacu.........................................................   6    0   JCK-1
.........................uuguagacgcuguguggac............................................................   5    0   LT-2
.........................uuguagacgcuguguggacac..........................................................   1    0   LCK-2
.........................uuguagacgcuguguggacacuccugc....................................................   3    0   LCK-3
............................uagacgcuguguggacacuccugc....................................................   1    0   LT-1
..............................................................cuucacgaugucuucaaguuag.....................   1    0   JCK-2
..............................................................cuucacgaugucuucaaguuagc....................   1    0   JCK-3
..............................................................cuucacgaugucuucaaguuagc....................   1    0   JCK-1
..............................................................cuucacgaugucuucaaguuagcU...................   1    1   JCK-1
..............................................................cuucacgaugucuucaaguuagcU...................   3    1   JCK-2
..............................................................cuucacgaugucuucaaguuagca...................   2    0   JCK-2
................................................................ucacgaugucuucaaguuag.....................   1    0   JCK-1
................................................................ucacgaugucuucaaguuagcU...................   1    1   JCK-3
.......................................................................................ccucuuuuuugcaucaug   1    0   LT-3
```
